# Decline of Alcohol as a Medicine

**Published:** 1896-04

**Authors:** T. D. Crothers

**Affiliations:** Hartford, Conn.


### Decline of Alcohol as a Medicine.

BY T. D. CROTHERS, M.D., HARTFORD, CONN.

Thoughtful observers recognize that alcohol as a medicine is rapidly becoming a thing of the past. Ten years ago leading medical men and text-books spoke of stimulants as essentials of many diseases, and defended their use with warmth and positiveness. To-day this is changed. Medical men seldom refer to spirits as remedies, and, when they do, express great conservatism and caution. The text-books show the same changes, although some dogmatic authors refuse to recognize the change of practice, and still cling to the idea of the food value of spirits.

Druggists who supply spirits to the profession recognize a tremendous dropping off in the demand. A distiller who ten years ago sold many thousand gallons of choice whiskies almost exclusively to medical men, has lost his trade altogether and gone out of business. Wine men, too, recognize this change, and are making every effort to have wine used in the place of spirits in the sick-room. Proprietary medicine dealers are putting all sorts of compounds of wine with iron, bark, etc., on the market with the same idea. It is doubtful if any of these will be able to secure any permanent place in therapeutics.

The fact is, alcohol is passing out of practical therapeutics because its real action is becoming known. Facts are accumulating in the laboratory, in the autopsy room, at the bedside and in the work of experimental psychologists, which show that alcohol is a depressant and a narcotic; that it cannot build up tissue, but always acts as a degenerative power, and that its apparent effects of raising the heart's action and quickening functional activities are misleading and erroneous.

French and German specialists have denounced spirits both as a beverage and a medicine, and shown by actual demonstration that alcohol is a poison and a depressant, and that any therapeutic action it is assumed to have is open to question.

All this is not the result of agitation and wild condemnation by persons who feel deeply the sad consequences of the abuse of spirits. It is simply the outcome of the gradual accumulation of facts that have been proven within the observation of every thoughtful person. The exact or approximate facts relating to alcohol can now be tested by instruments of precision. We can weigh and measure the effects, and it is not essential to theorize or speculate; we can test and prove with reasonable certainty what was before a matter of doubt.

Medical men who doubt the value of spirits are no more considered fanatics or extremists, but as leaders along new and wider lines of research. Alcohol in medicine, except as a narcotic and anæsthetic, is rapidly falling into disfavor, and will soon be put aside and forgotten.—*Exchange.*

